# Monthly Summary

**Published:** 1890-10

**Authors:** 


### Monthly Summary.

DANGERS OF HYPNOTISM.—The dangers of hypnotism are, according to the *Lancet*, not a few. At Nurembury a case of some public interest has recently been tried in the police court. A commercial traveller, while in a restaurant, told the waitress to look steadily at the white of his eye and hypnotized her. On the second occasion he repeated the experiment, but this time the sleep was so profound that a medical man had to be called, who had the utmost difficulty in rousing the girl. The commercial traveller was accordingly summoned to appear before the magistrates, and the severe sentence of eight days' imprisonment was passed on him, which will probably be efficient in checking similar performance in that region. In France the practice of hypnotizing people for amusement seems very common, and unpleasant consequences are reported. At a supper party in Paris recently one of the company hypnotized a girl and was unable to rouse her. She was consequently taken to the house of a medical man, and after a time she recovered consciousness. The whole party was taken in custody by the police, and were not released until next day. Even when hypnotism has been practiced by competent medical men for remedial purposes, unpleasant accidents and ulterior consequences have again and again occurred, so much so, that recently an order has been issued by the French Government prohibiting surgeons in the army and navy from practicing it. It ought to be distinctly understood, both by the profession and the public, that hypnotism is not devoid of danger at the time, and not infrequently has permanently impaired the moral and emotional control of patients. A medical man is bound, before recommending hypnotism for a patient, to weigh the question as carefully as he would that of the advisability of administering an anæsthetic.—*London Dental Record.*

DANGER IN EXERCISE.—The *Providence Journal* quotes Dr. Patton, Chief Surgeon of the National Soldiers' Home at Dayton, Ohio, as saying in an interview in Pittsburgh, the other day, that of the 5,000 soldiers in the Dayton home "fully 80 per cent. are suffering from heart disease in one form or another, due to the forced physical exertion of the campaign." And he made the prediction that as large a percentage of the athletes of to day will be found 25 years from now to be victims of heart disease, resulting from the muscular strains that they force themselves to undergo. As for the likelihood of exercise to prolong life, it may be said that according to the statistics of M. de Solaiville there are more people living in France to-day who have passed the age of 60 than there are in England, the home of athletic sports. And there is probably no nation in Europe more adverse to muscular cultivation for its own sake than the French. Great athletes die young, and a mortality list of Oxford rowing men published a few years ago showed that a comparatively small percentage of them lived out the allotted lifetime. Dr. Jastrow has demonstrated in some very elaborate statistics that men of thought live on an average three and a half years longer than men in the ordinary vocations of life.

---

PYORRHEA ALVEOLARIS COMPLICATED WITH NECROSIS.—*By Dr. John Coyle.*—My paper is a brief one—confined in fact to the relation of a case from practice which I think will prove interesting, if not instructive. In February a patient presented, and an examination showed that the cause of his suffering was the right inferior incisor. It was a distinct case of pyorrhea alveolaris, pus exuding from the pocket. This was the only tooth in his mouth which was affected. No calculus was present either on the affected tooth or any others. It was evidently a case beyond hope, and extraction was resorted to. It is generally claimed that after extraction the usual course is a restoration of the parts to health. Indeed, this is one of the arguments used by those who claim that this is solely a local disturbance.

In March the patient returned. Examination showed that necrosis had supervened, involving the alveolar ridge from the molar on the left side to the canine on the right. All the teeth were excessively loose, though they had been perfectly firm when last seen. The treatment was as follows: The teeth were ligated together, thus making them somewhat firm, and the diseased parts washed daily with a mixture of acetate of zinc—one drachm to the ouuce of water. As a tonic, the following was prescribed:

R

| | | |
|---|---|---|
| Potass. Iodidi, | - - - - | oz j |
| Tinct. Gentian Comp., | - - - | " iv |
| Sprts. Frumenti, | - - - - | " xii |

M. Sig.—Tablespoonful three times daily.

In October all the loosened teeth were firm in place and the disease was controlled. A point of interest is that the necrosis was confined to the cancelous portion of the bone only.—*Dental Mirror*.

---

HYPODERMIC INJECTIONS OF ERGOT IN FACIAL NEURALGIA.—Dr. Stewart, in *Peoria Medical Monthly*, says: For the relief of facial neuralgia hypodermic injections of ergot are far superior to either aconite or gelsemium. Any one who has used it will never resort to either of the above named remedies. I have used it in the last six years and have never had it fail in but one case. In that case there was evidently organic disease. Ordinarily one injection relieves the pain permanently; sometimes two; and in one very severe and obstinate case, which had gone through the hands of several physicians without relief, it required three. After the third injection he never had a twinge of pain. I put it in the temple as nearly over the seat of pain as convenient. I used the plain extract, and have it made on purpose for hypodermic use. One minim represents two grains of ergot. Of this I use from eight to twelve minims of blood warm at one injection, and without diluting. In order to make this a success two things are essential; one is, to have a fresh and pure article of ergot to make the extract from; the other is, to have the extract reasonably fresh. If kept too long it is not only worthless but irritating. When properly prepared and fresh it produces more or less pain for ten or fifteen minutes, and when the pain from the injection subsides the neuralgia is usually gone and does not return. I have used this treatment for sciatica and other forms of neuralgia, but not with very satisfactory results.—*Buffalo Medical and Surgical Journal.*

---

A PRACTICAL METHOD OF ELECTRO-GILDING GOLD DENTURES, BRIDGE-WORK, AND COLLAR CROWNS.—Dr. H. Fielden Briggs in *London Dental Record* describes his method as follows: First prepare in the following manner a stock solution of gilding fluid. (1) Take of pure gold 30 grains and digest in aqua regia ($HNO_3 + 3HCl$.); (2) evaporate *almost* but not quite to dryness; (3) dissolve this in twenty ounces of water; (4) then add half ounce of cyanide of potassium. This fluid will last a long while, and should be kept in a bottle ready for future use at any time.

*To Gild:* Heat gilding solution in jar in saucepan of water to about 150° Fah. While this is heating, polish the denture with whiting, wash well with plenty of soap, and place it into a basin of clean water; then avoid handling or exposing it to the air.

Attach to the positive electrode a thin sheet of fine gold, which should be not less in area than the piece to be gilded.

To the negative electrode attach the denture. When the gilding solution is heated, place the positive and negative electrodes with their attachments into it.

In a few minutes a dull brownish yellow deposit will be found on the denture. Polishing on the lathe with whiting will produce a rich deep gold appearance, giving the plate uniformity of color, obscuring the distinctness between it and the solder, and giving a perfectly finished aspect which lasts for many years.

By using a battery of six (1 quart) Leclanche cells and keeping the sheet of gold always attached to the positive electrode, a piece can be gilded at any time in a very few minutes.—*Dental Record.*

